# Retrospective Analysis of Antibiotic Use in Dogs with Chronic Inflammatory Enteropathy Prior to Referral: 144 Cases (2020–2024)

**DOI:** 10.3390/ani16121763

**Published:** 2026-06-08

**Authors:** Amy Norman, Tim Sparks, Matthew Best, Gerard Olivares, Michelle Walton-Clark

**Affiliations:** 1 Anderson Moores Veterinary Specialists, Part of Linnaeus Veterinary Limited, The Granary, Bunstead Barns, Poles Lane, Hursley, Winchester SO21 2LL, UK; 2 Eastcott Referrals, Part of Linnaeus Veterinary Limited, Edison Business Park, Hindle Way, Dorcan Way, Swindon SN3 3FR, UK; 3 Waltham Petcare Science Institute, Freeby Lane, Waltham on the Wolds, Melton Mowbray LE14 4RT, UK

**Keywords:** metronidazole, amoxicillin-clavulanate, diarrhoea, antimicrobial resistance, intestinal microbiome, antibiotic-responsive enteropathy

## Abstract

Chronic inflammatory enteropathy (CIE) has historically been divided into food-responsive chronic inflammatory enteropathy (CIE-FR), immunosuppressant-responsive chronic inflammatory enteropathy (CIE-IR), non-responsive chronic inflammatory enteropathy (CIE-NR) and antibiotic-responsive chronic inflammatory enteropathy (CIE-AR). Given the rising concern for antimicrobial resistance (AMR) and the effects of antibiotics on the microbiome, replacement of CIE-AR with microbiota-modulation-responsive enteropathy (MrMRE) has been suggested, and antibiotic use questioned. The objective of this study was to assess the prescribing habits of antibiotics for dogs with CIE prior to referral. This was a single-centre retrospective study of cases between 2020 and 2024 assessing antibiotic prescription for CIE in the six months prior to referral. Data including signalment, clinical signs, diagnosis, antibiotic administration and the antibiotics used were recorded. A total of 144 dogs with chronic inflammatory enteropathy were included, of which 128 had CIE without protein-losing enteropathy (PLE) and 16 had PLE. Overall, 57.6% of dogs with CIE referred for investigation were prescribed antibiotics in the six months prior to referral. Of the dogs prescribed antibiotics, metronidazole was the most prescribed (74.7%), followed by amoxicillin-clavulanate (36.1%). Dogs with diarrhoea were more likely to be prescribed antibiotics overall and were also more likely to receive metronidazole. Antibiotic use differed across the years, with the lowest rate prescribed in 2024 (26/46 [56.5%] in 2020/2021, 24/33 [72.7%] in 2022, 22/35 [62.9%] in 2023, and 11/30 [36.7%] in 2024; *p* = 0.031). Further work is required to understand continued antibiotic use in CIE and inform antimicrobial stewardship strategies.

## Simple Summary

Long-term gastrointestinal disease is a common problem in dogs and can be challenging to manage. Traditionally, dogs with this condition have been grouped based on whether they improve with diet changes, medications that control the immune system, or antibiotics. However, the use of antibiotics for this condition is now being questioned, and alternative approaches are increasingly recommended. In this study, we reviewed the records of dogs referred to a specialist hospital for investigation of long-term gastrointestinal disease and recorded whether they had received antibiotics in the six months before referral. We found that over half of the dogs (57.6%) had been treated with antibiotics before being referred. This suggests that antibiotics are commonly prescribed for this condition before referral despite increasing evidence that their use may not always be beneficial. Encouragingly, we also found that the lowest antibiotic prescription rate was in the most recent study year. Antibiotics are essential for treating serious infections in both animals and people, but their overuse can lead to antibiotic resistance, making infections harder to treat. This study highlights the importance of using antibiotics carefully and supports ongoing efforts to improve treatment strategies for dogs with long-term diarrhoea.

## 1. Introduction

Chronic inflammatory enteropathy (CIE) is characterised by chronic (>3 weeks) gastrointestinal signs commonly including vomiting, diarrhoea, weight loss, hyporexia or nausea [1]. It is estimated to affect 0.9–2% of dogs presenting to referral veterinary hospitals per year [2,3]. CIE is currently classified by the response to treatment, resulting in the categories food-responsive chronic inflammatory enteropathy (CIE-FR), immunosuppressant-responsive chronic inflammatory enteropathy (CIE-IR) and antibiotic-responsive chronic inflammatory enteropathy (CIE-AR) [4]. Those that do not respond to treatment are known as non-responsive chronic inflammatory enteropathy (CIE-NR) [4]. Additionally, CIE that results in hypoalbuminaemia is described as a protein-losing enteropathy (PLE) [5].

The exact pathogenesis of CIE is still not known, but the most accepted hypothesis is an interplay of genetic predisposition, environmental factors, altered intestinal microbiome, altered mucosal immune response and epithelial barrier dysfunction [6]. CIE-FR is the largest subcategory of CIE with approximately 50–65% of canine cases expected to respond to dietary management [1,4,7,8,9]. Dietary modification is thought to have beneficial effects on the intestinal microbiome diversity and function, intestinal motility, barrier function and regulation of the immune system [8,9,10]. One study found that approximately two-thirds of CIE-NR cases responded favourably to an alternative diet trial and 90% of CIE-NR had improvement in their Canine Inflammatory Bowel Disease Activity Index (CIBDAI) score [9]. This suggests that some patients may require multiple diet trials to achieve clinical remission and prevent misclassification as an NRE [7].

More recently, the role of antibiotics in CIE has been scrutinised [7]. Although a vital and potentially lifesaving intervention, antibiotics also affect the diversity of the gastrointestinal microbiome [11,12], leading to significant alterations [1,13]. The use of antibiotics is known to have a deleterious impact on the intestinal microbiota, with long-lasting effects [11,14,15,16,17,18]. Intestinal dysbiosis in humans has been associated with the use of systemic antibiotics, which has been shown to increase the risk of the development of irritable bowel disease (IBD), asthma, obesity and coeliac disease [19,20,21]. CIE cases classified as CIE-AR have historically been challenging to manage. They are frequently shown to have high relapse rates, with one prospective study showing that dogs that responded to tylosin had a relapse rate of 85.7% within 30 days of discontinuation of their antibiotic therapy [22]. In addition, tylosin has been associated with intestinal microbiome disruption and alterations to faecal unconjugated bile acids [23]. Given that a proportion of cases classified as CIE-NR are misclassified and require further diet trials, it has been questioned whether the CIE-AR group is similar and would in fact respond to dietary management following multiple trials [1,7]. It has therefore been proposed that CIE-AR should be replaced by the category microbiota-related-modulation-responsive enteropathy (MrMRE) [10], and strategies to modulate the intestinal microbiota (diet, prebiotics, probiotics, faecal microbiota transplantation (FMT)) should be considered in place of antibiotic administration [7,10].

Despite antibiotic stewardship schemes and tools, a significant number of patients presenting with varying diseases have been shown to receive antibiotics prior to referral against the stewardship guidelines [24]. Drivers of inappropriate antibiotic prescriptions have previously been linked to factors such as client satisfaction and retention, commercial pressures, influence of senior colleagues, limited client knowledge of antimicrobial resistance (AMR), cost of culture and sensitivity and treating ‘just in case’ [25,26]. However, despite these perceived pressures, owners have been shown to value a clear plan and the experience of their veterinarian, and they did not appear to expect antibiotic prescriptions [27]. Another potential factor that could influence antibiotic prescription is that CIE can cause significant impacts on the quality of life for both dogs and their owners, leading to caregiver burden [28], which may in turn increase the perceived urgency of veterinarians to intervene.

Antibiotic use in companion animals has been associated with the selection of antimicrobial-resistant bacteria, contributing to a growing global health concern [29,30]. Although most of the data on AMR is from the human literature, antibiotic use has significant effects on both human and animal health [29,31]. It was estimated that in 2019 that there were 4.95 million human deaths associated with drug-resistant infections, with 1.27 million deaths directly attributed to AMR [31]. Predicted mortality rates for AMR by 2050 are forecasted to increase to 1.91 million attributable deaths and 8.22 million associated deaths annually [32]. In addition to the risk posed to human health, AMR is also detrimental to animal health as high rates of treatment failure may lead to poor outcomes and potentially euthanasia [29].

The aim of the current study was to describe the antibiotic prescribing habits prior to referral in dogs with chronic inflammatory enteropathy and to assess the trends over a five-year period. We hypothesised that antibiotic prescribing would reduce over the years as we gained further understanding of MrMRE and moved away from CIE-AR.

## 2. Materials and Methods

This was a retrospective, single-centre study enrolling dogs investigated for suspected CIE. The electronic database was searched for dogs that underwent abdominal ultrasonography between January 2020 and December 2024 which was undertaken by an ECVIM-boarded diagnostic imaging specialist or a CertAVP in diagnostic imaging supervised by a diplomate. A diagnosis of CIE was based on compatible chronic gastrointestinal clinical signs (gastrointestinal signs for greater than three weeks’ duration, including one or more of the following: vomiting, diarrhoea, hyporexia, or weight loss) and exclusion of other identifiable gastrointestinal or extra-intestinal disease processes by the attending internal medicine clinician. Due to the retrospective nature of the study, diagnostic investigations were not standardised and were performed at the discretion of the attending clinician. Dogs were classified as having PLE if hypoalbuminaemia was present in conjunction with clinical findings supportive of gastrointestinal protein loss. Hypoalbuminaemia was defined as an albumin level below the lowest reference range of 22 g/L in conjunction with compatible gastrointestinal clinical signs.

Dogs were excluded from the study if investigations revealed the presence of other gastrointestinal diseases (infectious diarrhoea, gastrointestinal foreign bodies, exocrine pancreatic insufficiency, gastrointestinal neoplasia) or extra-intestinal causes of gastrointestinal signs (including endocrinopathies, non-gastrointestinal neoplasia, infectious disease), or if there was an incomplete history for the six-month period prior to referral.

Incomplete medical history was defined as insufficient documentation regarding clinical signs, previous diagnostic investigations, or antibiotic administration during the six months prior to referral. Classification of gastrointestinal signs, including small bowel or large bowel diarrhoea, was based on the clinical history documented within the medical records and referral documentation. Antibiotic exposure was defined as documented antimicrobial prescription or reported administration within the six months prior to referral for gastrointestinal clinical signs consistent with chronic enteropathy. This information was obtained from referring veterinary records and referral histories.

The following information was retrieved from the records: signalment, weight at referral, body condition score at referral (BCS), date and reason for first presentation to the referral centre, nature of clinical signs (large bowel diarrhoea, small bowel diarrhoea, mixed bowel diarrhoea, vomiting, retching, weight loss, hyporexia, bloating, borborygmi, regurgitation, anorexia and melena), and a clinical diagnosis of CIE or PLE based on compatible clinical signs and exclusion of other causes. Antibiotic administration for clinical signs consistent with chronic enteropathy in the six months prior to referral was recorded from the referring veterinarians’ clinical histories, together with the specific antibiotic agents used. Due to COVID-19, the case numbers in 2020 were small, and so 2020 and 2021 were assessed together.

### Statistical Analysis

Data were incomplete for some variables, and analysis was based on all available data. Continuous variables were compared between two groups using Mann–Whitney U tests adjusted for ties. Comparisons between categorical variables were made using chi square tests of association or Fisher’s exact tests if counts were low. Because of low numbers in 2020, data for 2020 and 2021 were combined for analysis. A multiple binary logistic regression of antibiotic use on those complete independent variables that generated univariate *p*-values < 0.20 was undertaken with backwards elimination of nonsignificant variables. All analysis was undertaken in Minitab Version 22. Significance was taken as *p* < 0.05.

## 3. Results

Records of 982 dogs were screened for inclusion, and 144 dogs met the inclusion criteria. A total of 838 cases were excluded for the following reasons: neoplasia (137), hepatobiliary disease (116), immune-mediated disease (103), incomplete records (62), acute gastrointestinal disease (58), renal disease (57), urinary disease (55), endocrinopathies (43), gastrointestinal foreign body (38), pancreatic disease (23), infectious disease (22), neurological disease (20), sepsis (18), other non-gastrointestinal disease (17), reproductive disease (16), respiratory disease (12), surgical disease (11), vascular abnormalities (10), electrolyte disturbances (7), splenic disease (5), anaphylaxis (5), and toxins (3).

The most common breeds included crossbreed (24/144 [16.7%]), Labrador Retriever (11/144 [7.6%]), and English Cocker Spaniel (10/144 [6.9%]). Other breeds included Jack Russell Terrier (8/144 [5.6%]), Border Terrier (7/144 [4.7%]), French Bulldog (6/144 [4.2%]), English Springer Spaniel (6/144 [4.2%]), Miniature Schnauzer (5/144 [3.5%]) and Dachshund (5/144 [3.5%]). Forty other breeds were represented by between 1 and 4 dogs (see Supplementary Information, Table S1).

Of the 144 dogs included in the study, 128 (88.9%) were diagnosed with CIE without PLE and 16 (11.1%) with PLE. Among the study dogs, the median age at the time of the initial evaluation was 5.6 years (range 0.5 to 14.4 years). There were 77 (53.5%) males (40 [27.8%] entire and 37 [25.7%] neutered) and 67 (46.5%) females (9 [6.4%] entire and 58 [40.3%] neutered). Median weight was 10.7 kg (range 2.0 to 50.2 kg), with a median BCS of 4/9 (range 1 to 7). Of the dogs in the study, 5/144 (3.5%) were seen in 2020, 41/144 (28.5%) were seen in 2021, 33/144 (22.9%) were seen in 2022, 35/144 (24.3%) were seen in 2023, and 30/144 (20.8%) were seen in 2024.

**Figure 1:**
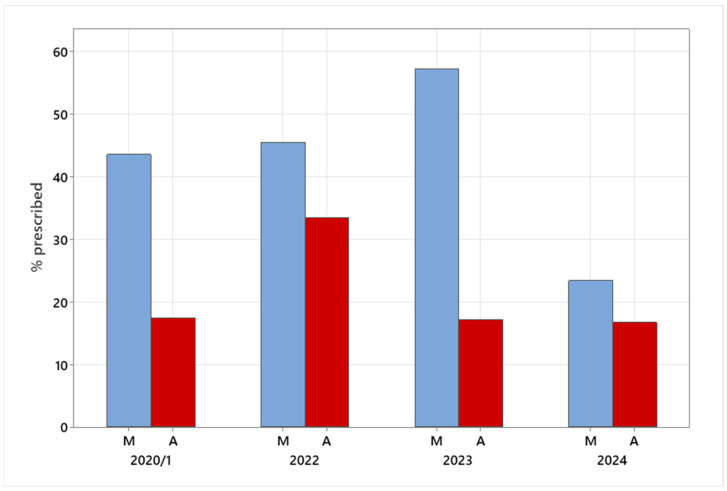
Prescription rates of amoxicillin-clavulanate (A) and Metronidazole (M) 2020/1–2024.

**Figure 2:**
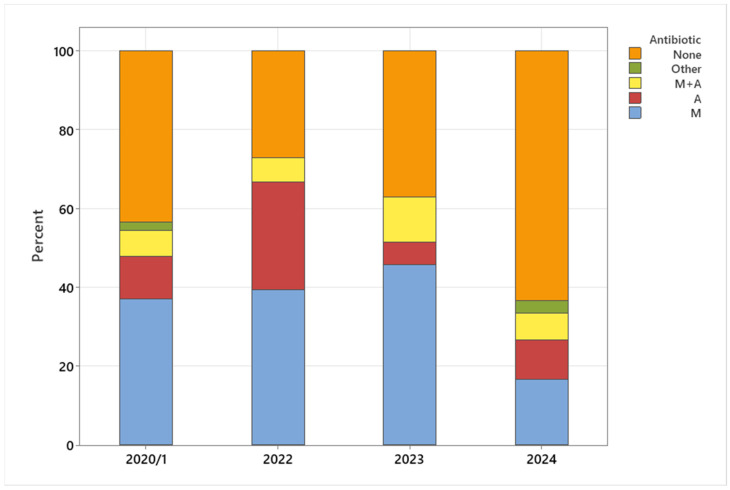
Antibiotic prescription in 2020/1–2024 for CIE in the six months prior to referral (M: metronidazole; A: amoxicillin-clavulanate; M + A: both metronidazole and amoxicillin-clavulanate; Other: single antibiotic other than M or A). Three of the M cases, one of the A cases and one of the M + A cases included an additional antibiotic.

Antibiotics were prescribed prior to referral in 83/144 (57.6%) of dogs. The signalment of dogs which did and did not receive antibiotics is summarised in Table 1. There was no significant difference in age, weight, BCS or sex/neuter status between groups. Antibiotics were prescribed to 71/128 (55.6%) dogs with CIE and 12/16 (75.0%) dogs with PLE; this difference was not significant. Of the dogs prescribed antibiotics, metronidazole was most commonly prescribed (62/83 [74.7%]), followed by amoxicillin-clavulanate (30/83 [36.1%]). This was a consistent trend across all years, as shown in Figure 1. Other antibiotics prescribed were used in single cases (each 1/83 [1.2%] of dogs receiving antibiotics); these were: enrofloxacin, doxycycline, marbofloxacin, cefovecin, amoxicillin, oxytetracycline and erythromycin. In the period prior to referral, 68/144 (47.2%) dogs had received one antibiotic, 14/144 (9.7%) had received two different antibiotics, and 1/144 (0.7%) had received three. Antibiotic prescribing varied by year, with the highest prescribing observed in 2022 and the lowest in 2024 (Figure 2); there was a significant difference in antibiotic prescription across the years studied (*p* = 0.031).

Dogs presenting with PLE were more likely to be treated with amoxicillin-clavulanate (7/16; 43.7%) than those with CIE (23/128; 18.0%) (*p* = 0.044). When diarrhoea types were combined (small intestinal, large intestinal and mixed), dogs with diarrhoea were more likely to be prescribed antibiotics than those without diarrhoea (68/104; 65.4% vs. 14/39; 35.9%, *p* = 0.002). In addition, dogs with diarrhoea were more likely to be treated with metronidazole than those without diarrhoea (54/104; 51.9% vs. 7/39; 17.9%, *p* < 0.001).

A multiple binary logistic regression of antibiotic use with backward elimination on year, sex, diagnosis (CIE or PLE) and combined diarrhoea yielded two significant variables: a positive association with diarrhoea (*p* = 0.002) and year differences (*p* = 0.048), with 2024 lowest.

## 4. Discussion

The results of the current study show that antibiotics were prescribed for 57.6% of dogs with CIE in the six months prior to referral. This is similar to a previous study which reported that 53% of dogs and cats were prescribed antibiotics prior to referral, although this was not specific to gastrointestinal disease [24]. This study also highlighted that antibiotic prescribing was frequently empirical, with limited diagnostic testing prior to administration [24]. In the current study, metronidazole was the most prescribed antibiotic (74.7%), followed by amoxicillin-clavulanate (36.1%). Similar findings have been reported previously in dogs with acute diarrhoea, where metronidazole was prescribed in 72% and amoxicillin-clavulanate in 28% of cases [33].

There was a significant difference in antibiotic prescription across the years studied (*p* = 0.031), with the lowest prescribing observed in 2024 (36.7%). This may reflect changing prescribing practices and increasing awareness of antimicrobial stewardship. However, as only five years of data were assessed and relatively few cases were included in 2024, these findings should be interpreted cautiously. Despite the reduction in prescribing, over one-third of cases were still prescribed antibiotics for CIE, most commonly metronidazole. This is likely due to the historical categorisation of CIE-AR and the perceived immunomodulatory effects of metronidazole [34]. Interestingly, antibiotic prescription increased from 2020/2021 (56.5%) to 2022 (72.7%). The lower prescribing rates in 2020/2021 may have reflected COVID-19-related restrictions, which may have prompted earlier referral for investigation rather than reliance on treatment trials in primary care.

Metronidazole was the most prescribed antibiotic in the current study (74.7% of dogs receiving antibiotics), a finding supported by a Swedish study in which metronidazole was the most frequently prescribed antibiotic in dogs with CIE (95.3%) [3]. In addition, metronidazole was significantly more likely to be prescribed in our study for dogs presenting with diarrhoea than those without (54/104; 51.92% vs. 7/39; 17.95%, *p* < 0.001), suggesting a perceived benefit of its use in the management of diarrhoea. Metronidazole is commonly prescribed for chronic diarrhoea, with many clinicians reporting its use for perceived anti-inflammatory or immunomodulatory effects [34]. However, evidence supporting these benefits remains inconsistent within the veterinary literature [35]. Previous studies suggested that metronidazole may have direct immunomodulatory effects [36,37]. However, subsequent studies have suggested that these effects may instead be secondary to alterations in the intestinal microbiome rather than a direct anti-inflammatory mechanism [38,39]. Metronidazole administration has been shown to alter the intestinal microbiome, including reductions in *Peptacetobacter hiranonis*, a bacterial species associated with bile acid metabolism and considered a marker of canine dysbiosis, alongside increased detection of potentially pathogenic and antimicrobial-resistant bacterial strains [40,41,42,43].

Another possible reason for metronidazole prescription is the belief that it may shorten the duration of diarrhoea, as has been suggested in dogs with acute diarrhoea [44]. However, that study was limited by a relatively small sample size, a short follow-up period, and restriction to dogs with uncomplicated acute diarrhoea, limiting extrapolation to dogs with CIE and referral populations. In contrast, several studies have shown no significant difference in disease duration or severity between metronidazole and nutraceutical therapies in dogs with gastrointestinal disease [40,45].

The second-most-prescribed antibiotic in the current study was amoxicillin-clavulanate (36.1% of cases prescribed antibiotics). Amoxicillin-clavulanate is a broad-spectrum antibiotic [46] classified as a highly important antibiotic for human health by the World Health Organisation (WHO) [47]. Despite antimicrobial stewardship concerns, β-lactamase inhibitor combinations, predominantly amoxicillin-clavulanate, represent a large proportion of veterinary antimicrobials used in Europe [29]. The reason for its prescription in the current study population is unclear. The PROTECT ME guidelines are an antimicrobial stewardship initiative and directive developed by the British Small Animal Veterinary Association (BSAVA) to help guide the rational and responsible use of antimicrobial prescribing within veterinary practice [48]. These guidelines recommend that amoxicillin-clavulanate be used primarily in cases of acute diarrhoea associated with systemic inflammatory response syndrome (SIRS)/sepsis or specific infectious causes [48,49]. Previous studies have also associated amoxicillin-clavulanate administration with increased detection of resistant *Escherichia coli* isolates and alterations in the intestinal microbiome [30,50]. Given the limited evidence supporting its use in CIE, further work is needed to better understand the reasons for its continued prescription in these cases.

Antibiotics were prescribed to 71/128 (55.6%) dogs with CIE, and 12/16 (75.0%) dogs with PLE, although this difference was not significant. However, this may reflect the relatively small number of dogs with PLE included in the current study. We found that PLE cases were significantly more likely to receive amoxicillin-clavulanate than dogs with CIE alone (7/16; 43.7% vs. 23/128; 18.0%, *p* = 0.044). Dogs with PLE may present with more severe clinical signs, hypoalbuminaemia, and marked intestinal lesions on endoscopic biopsies [51,52,53]. Loss of mucosal integrity in these cases may increase concern regarding bacterial translocation and sepsis, although the evidence supporting this remains limited [5]. These factors may explain both the higher frequency of antibiotic administration and the preferential use of amoxicillin-clavulanate in dogs with PLE.

In the current study, age, weight, sex, and body condition score were not associated with antibiotic prescribing. Previous studies assessing antimicrobial prescribing across a broader range of conditions have identified associations between antibiotic prescription and factors including age, weight, vaccination status, insurance status, and neuter status [54,55,56]. Younger dogs have previously been shown to have increased odds of receiving antibiotics [54], potentially reflecting a greater perceived likelihood of infectious disease within this population. In contrast, other studies have reported that age and weight may influence the type of antibiotic prescribed rather than the overall likelihood of prescription [55]. Differences between these findings and the current study may reflect the exclusion of infectious diseases and the specific focus on dogs with CIE. Vaccination and insurance status have previously been associated with antimicrobial prescribing practices, but these were not assessed in the current study [56].

Antibiotic administration has been associated with the detection of antimicrobial-resistant bacteria [17,30]. Dogs treated with tylosin have been shown to have decreased diversity of the intestinal microbiome alongside increased numbers of tylosin-resistant *E. coli* and *Clostridium perfringens* [17]. Similar findings have been reported in other studies demonstrating increased numbers of antimicrobial-resistant bacteria following antibiotic administration, particularly resistant *E. coli* strains in canine faeces [13,30].

This study has several limitations. First, it was a retrospective, single-centre study and relied on referring veterinary histories and documentation of previous treatments. Although records were reviewed carefully, it is possible that some information regarding prior antibiotic therapy and treatment indications may have been incomplete or inaccurately documented, particularly when managed by multiple veterinarians prior to referral. This may have resulted in misclassification of antimicrobial exposure. Antimicrobial dosage, duration, and repeated courses were also not consistently documented and therefore could not be reliably assessed.

Prescribing practices may differ between individual clinicians and practices, which may have introduced bias into the results, although this was not assessed in the current study. The overall sample size was relatively small, particularly for dogs with PLE, which represented a smaller subgroup and may have limited the ability to detect significant associations within this population.

As this study was conducted in a referral population, the findings may not fully reflect antimicrobial prescribing practices within the wider primary care veterinary population. The retrospective design and lack of a standardised diagnostic investigation protocol may have resulted in heterogeneity within the study population. Dogs referred for specialist investigation may represent more complex or severe cases, potentially influencing treatment decisions prior to referral. Furthermore, the indication for antimicrobial administration may not always have been clear from the available clinical histories. Cases were identified through abdominal ultrasound records, which may have inadvertently excluded dogs with less severe or food-responsive disease that improved following dietary management alone, potentially biasing the study population toward more severe or treatment-refractory cases.

## 5. Conclusions

This study demonstrated that over half of dogs presented for investigations into CIE had been prescribed antibiotics prior to referral, with metronidazole being the most frequently prescribed antibiotic. Although a reduction in antibiotic prescribing was observed in 2024, the relatively small number of cases included during this period means that this finding should be interpreted cautiously. Further monitoring over a longer period would be required to determine whether this represents a sustained change in prescribing patterns. Increasing evidence suggests that many cases of CIE may be diet-responsive but may require multiple diet trials [7,8,9]. The findings of the current study highlight that antibiotics continue to be prescribed despite growing evidence suggesting limited clinical benefit in many cases and growing concerns regarding antimicrobial stewardship, intestinal microbiome disruption, and antimicrobial resistance [13,17,29,30,35]. Further research investigating alternative management strategies, including dietary modulation of the intestinal microbiome, prebiotics, probiotics, and faecal microbiota transplantation, may help improve management of CIE while reducing unnecessary antimicrobial use.

## Figures and Tables

**Table 1:** Signalment of dogs that did and did not receive antibiotics prior to referral. Weight was not recorded for 5 dogs, and BCS was not recorded for 64 dogs.

Variable		Dogs Receiving Antibiotics (*n* = 83)	Dogs Not Receiving Antibiotics (*n* = 61)	*p*
Age (years)	median (range)	6.5 (0.5–14.4)	5.3 (0.6–14.0)	0.280
Weight at referral (kg)	median (range)	10.0 (2.0–38.7)	12.5 (2.0–50.2)	0.792
Body condition score at referral (*n*/9)	median (range)	3.5 (1–7)	4.0 (2–7)	0.162
**Sex/neuter status**				
Female entire	*n* (%)	2 (2.4)	7 (11.5)	0.096
Female neutered	*n* (%)	32 (38.6)	26 (42.6)	
Male entire	*n* (%)	24 (28.9)	16 (26.2)	
Male neutered	*n* (%)	25 (30.1)	12 (19.7)	
**Diagnosis**				
Chronic inflammatory enteropathy	*n* (%)	71 (85.5)	57 (93.4)	0.136
Protein-losing enteropathy	*n* (%)	12 (14.5)	4 (6.6)	

## Data Availability

The data supporting the findings of this study are not publicly available due to privacy and ethical restrictions.

## Supplementary Materials

The following supporting information can be downloaded at: https://www.mdpi.com/article/10.3390/ani16121763/s1, Table S1: Breed distribution in CIE patients.

## Abbreviations

The following abbreviations are used in this manuscript:

AMR	Antimicrobial resistance
BCS	Body condition score
CIBDAI	Canine Inflammatory Bowel Disease Activity Index
CIE	Chronic inflammatory enteropathy
CIE-AR	Antibiotic-responsive chronic inflammatory enteropathy
CIE-FR	Food-responsive chronic inflammatory enteropathy
CIE-IR	Immunosuppressant-responsive chronic inflammatory enteropathy
CIE-NR	Non-responsive chronic inflammatory enteropathy
FMT	Faecal microbiota transplantation
MrMRE	Microbiota-modulation-responsive enteropathy
PLE	Protein-losing enteropathy
