# Supplementary material for: Retrospective Analysis of Antibiotic Use in Dogs with Chronic Inflammatory Enteropathy Prior to Referral: 144 Cases (2020–2024)
Source: Animals (Basel). 2026 Jun 8;16(12):1763. doi: 10.3390/ani16121763 (PMC13296299; doi:10.3390/ani16121763)
Supplement: Supplementary file 1 [file animals-16-01763-s001.zip › animals-4322476-supplementary.pdf]

Table 1 showing breed distribution for CIE dogs

Breed	Count	Percent
Afghan Hound	1	0.69
Bassett Hound	1	0.69
Bearded Collie	1	0.69
Bichon Frise	4	2.78
Border Collie	4	2.78
Border Terrier	7	4.86
Boston Terrier	2	1.39
Bulldog	1	0.69
Cavachon	1	0.69
Cavalier King Charles Spaniel	2	1.39
Cavapoo	1	0.69
Cocker Spaniel	10	6.94
Cockerpoo	4	2.78
Cross Breed	24	16.67
Dachshund	5	3.47
English Springer Spaniel	6	4.17
English Toy Terrier	1	0.69
Flat Coated Retriever	1	0.69
French Bulldog	6	4.17
German Shepherd	1	0.69
Golden doodle	1	0.69
Golden Retriever	3	2.08
Greyhound	1	0.69
Havanese	1	0.69
Jack Russell Terrier	8	5.56
Labradoodle	1	0.69
Labrador	11	7.64
Lurcher	2	1.39
Maltese	1	0.69
Miniature Dachshund	3	2.08
Miniature Poodle	2	1.39
Miniature Schnauzer	5	3.47
Norfolk Terrier	1	0.69
Nova Scotia Duck Tolling	1	0.69
Parsons Jack Russell Terrier	1	0.69
Pomeranian	2	1.39
Poodle	1	0.69
Pug	1	0.69
Schnauzer	1	0.69
Sheltie	1	0.69
Shih Tzu	2	1.39
Springador	1	0.69
Sprocker	2	1.39
Terrier	1	0.69
Tibetan Terrier	1	0.69
Vizsla	1	0.69
West Highland White Terrier	3	2.08
Whippet	1	0.69
Wire Fox Terrier	1	0.69
